# Case Report: Concurrent esophageal and spinal cord compression in cervical spondylosis: integrated anterior osteophytectomy and zero-profile ACDF for dual pathology decompression

**DOI:** 10.3389/fsurg.2025.1609708

**Published:** 2025-06-26

**Authors:** Jian Wu, Wei Shao, Wenqing Zhu, Jianwen Mo

**Affiliations:** ^1^ The First Clinical College, Gannan Medical University, Ganzhou, Jiangxi, China; ^2^ Department of Orthopedics, The First Affiliated Hospital of Gannan Medical University, Ganzhou, Jiangxi, China

**Keywords:** cervical spondylotic dysphagia, cervical spondylotic myelopathy, degenerative cervical disease, anterior cervical discectomy and fusion (ACDF), zero-profile interbody fusion devices, osteophyte and cervical disc herniation

## Abstract

Cervical spondylotic dysphagia (CSD) and cervical spondylotic myelopathy (CSM) represent two distinct clinical entities within degenerative cervical pathology. Their co-occurrence creates diagnostic and therapeutic dilemmas due to overlapping pathophysiological mechanisms. CSD primarily stems from anterior cervical osteophytes mechanically compressing the esophageal lumen, resulting in progressive dysphagia and pharyngeal discomfort. Conversely, CSM develops through spinal cord compression mediated by posterior osteophytic growth, intervertebral disc herniation, or ossification of the posterior longitudinal ligament (OPLL), clinically manifesting as limb paresthesia, motor weakness, gait instability, and impaired manual dexterity. We describe a 58-year-old male presenting with progressive dysphagia accompanied by bilateral lower extremity weakness. Radiological evaluation demonstrated prominent anterior osteophytes with bridging syndesmophytes at C4–C6 levels causing posterior pharyngeal wall displacement, concurrent with C3–C7 OPLL and multilevel disc herniations inducing spinal cord compression. Surgical management comprised anterior cervical osteophytectomy via a standard Smith-Robinson approach, followed by two-level anterior cervical discectomy and fusion (ACDF) utilizing a zero-profile interbody cage system, achieving dual objectives of spinal canal decompression and segmental stabilization. The patient exhibited complete dysphagia resolution and substantial neurological recovery during postoperative follow-up.

## Introduction

CSD has gained increasing clinical recognition as a distinct entity characterized by anterior osteophyte-induced esophageal compression. The disorder typically manifests with insidious-onset symptoms including pharyngeal foreign body sensation, progressive dysphagia, and voice changes, which are frequently misinterpreted as chronic laryngopharyngeal inflammation or functional gastrointestinal disorders (1, 2). CSM, the most prevalent spinal cord disorder in adults, arises from progressive cord compression secondary to degenerative alterations encompassing intervertebral disc collapse, posterior osteophyte formation, and ligamentous hypertrophy. This condition frequently leads to irreversible neurological deficits, substantially impairing patients' functional capacity and quality of life (3). The simultaneous presentation of CSD with CSM remains a rare clinical phenomenon, with limited documented cases in medical literature.

## Case presentation

**Figure 1:**
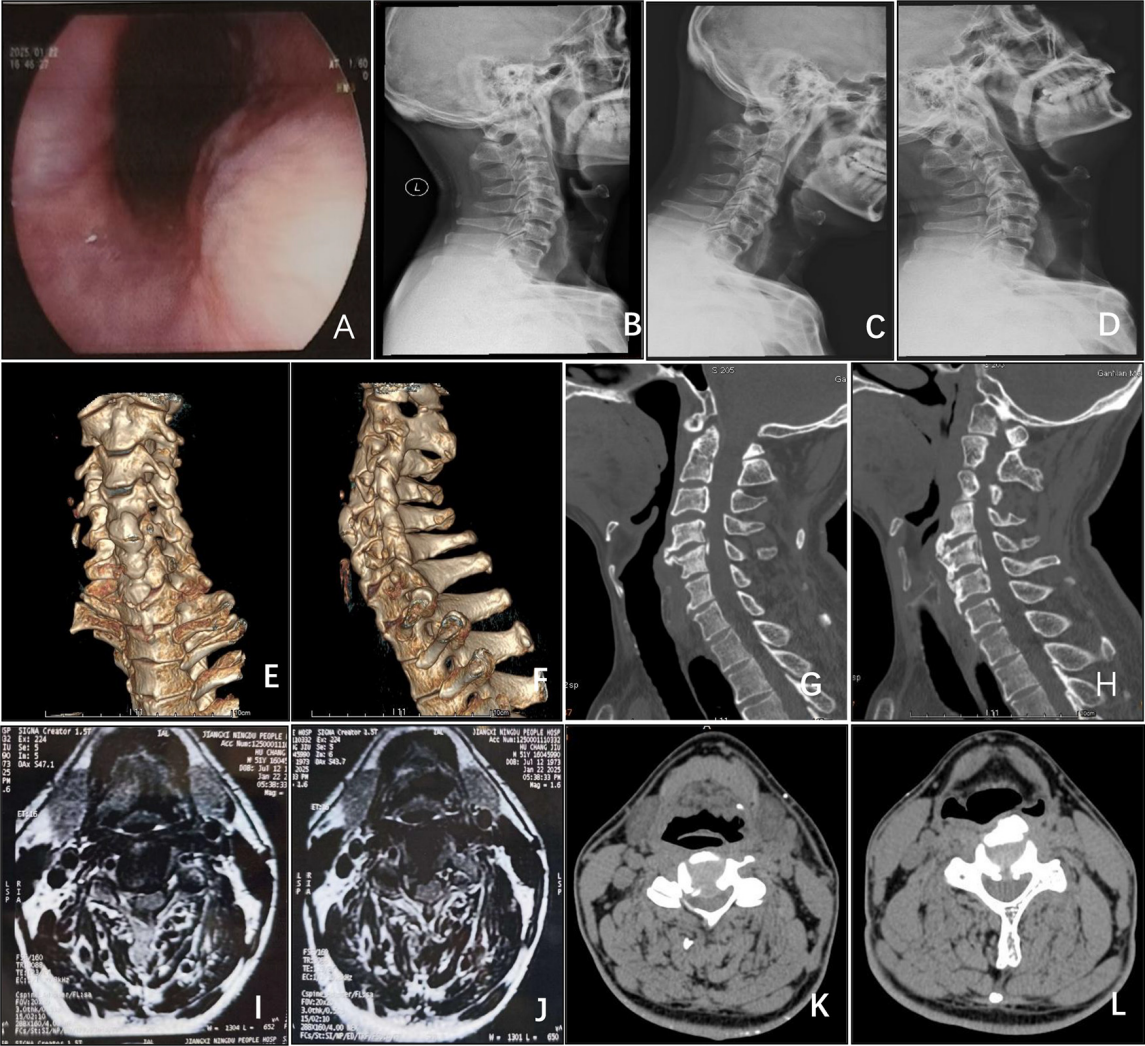
**(A)** Endoscopy reveals compression and protrusion of the esophageal mucosa, resulting in stenosis. **(B–D)** Lateral, flexion, and extension x-ray views of the cervical spine. **(E,F)** CT three-dimensional reconstruction of the cervical spine demonstrates large osteophytes on the anterior aspects of the C4 and C5 vertebral bodies. **(G,H)** Sagittal CT view of the cervical spine shows OPLL spanning C3–C7. **(I,J)** Axial MRI of the cervical spine and **(K,L)** axial CT of the cervical spine reveal disc herniation at C3/4 and C4/5, accompanied by posterior longitudinal ligament ossification compressing the spinal cord.

A 51-year-old male patient presented with a two-year history of progressively worsening dysphagia, which had significantly deteriorated over the preceding month. Previous treatment for suspected pharyngitis provided no meaningful relief. Concurrently, the patient reported six months of bilateral lower extremity weakness accompanied by a characteristic “walking on cotton” sensation, manifesting as slowed and unsteady gait during level walking, exacerbation of bilateral lower limb pain and numbness after approximately 100 meters of ambulation, and dependence on assistance for stair climbing. Neurological examination of the upper extremities revealed mildly reduced superficial cutaneous sensation, slightly hyperactive deep tendon reflexes, preserved muscle strength, and minimally delayed but functionally intact fine motor coordination. Lower extremity assessment demonstrated cutaneous numbness, grade V quadriceps strength, and mild weakness (grade IV) in tibialis anterior, ankle dorsiflexion, and great toe extension. Trunk sensation and bladder function remained intact. Positive Hoffmann signs were observed. Preoperative functional assessments yielded a Japanese Orthopaedic Association (JOA) score of 12, Neck Disability Index (NDI) of 66% (indicating severe disability), and Functional Oral Intake Scale (FOIS) level 4. Laboratory investigations, including complete blood count, C-reactive protein, thyroid function tests, and antinuclear antibody panel, returned normal results, effectively excluding inflammatory, autoimmune, or metabolic pathologies. Cervical ultrasonography revealed no thyroid enlargement or nodular lesions to suggest extrinsic compressive pathology. Abdominal ultrasound demonstrated no evidence of hiatal hernia or structural abnormalities at the gastroesophageal junction. The absence of heartburn, acid regurgitation, or unintended weight loss further argued against gastroesophageal reflux disease or malignancy. Additionally, the lack of meal-related symptom variability and negative history of neuromuscular disorders (e.g., myasthenia gravis) diminished the likelihood of primary esophageal dysmotility. Endoscopic evaluation identified pharyngeal wall protrusion with concomitant esophageal lumen narrowing (Figure 1A). Radiographic studies, including cervical plain films (Figures 1B–D), computed tomography (CT) scans (Figures 1G,H), and three-dimensional CT reconstructions (Figures 1E,F), demonstrated anterior osteophytic bridging at C4–6 vertebral levels with corresponding retropharyngeal displacement, OPLL spanning C3–7, and calcification of the ligamentum nuchae. Cervical magnetic resonance imaging (MRI) (Figures 1I,J) and CT myelography (Figures 1K,L) revealed posterior disc herniation at C3/4 and C4/5 levels, resulting in spinal cord compression.

### Surgical procedure

The patient underwent general anesthesia and was positioned supine with shoulder elevation and moderate cervical extension. Following standard sterile preparation and draping, a 7 cm vertical right anterior cervical incision was created. The surgical dissection proceeded sequentially through skin, subcutaneous tissue, and platysma muscle, accessing the plane between the carotid sheath laterally and visceral compartment medially adjacent to the sternocleidomastoid muscle. Meticulous blunt retraction of the tracheoesophageal complex and carotid neurovascular bundle exposed the anterior vertebral column. Intraoperative C-arm fluoroscopy with localization needle guidance confirmed the C3/4, C4/5, and C5/6 intervertebral levels. The prevertebral fascia was carefully dissected using peanut-shaped gauze, followed by incision of the hypertrophied anterior longitudinal ligament at C4–5 to expose the osteophyte base. A high-speed burr was systematically employed to remove osteophytes in a layered fashion from superficial to deep planes, fully exposing the anterior surfaces of C3, C4, and C5 vertebrae. Caspar retractors were then positioned to optimize operative exposure. Subsequent steps included complete excision of the C3/4 and C4/5 intervertebral discs and cartilaginous endplates using curettes, followed by meticulous resection of ossified posterior longitudinal ligament until complete dural sac decompression was achieved, effectively relieving thecal sac and nerve root compression. Autologous bone fragments harvested from osteophyte resection and vertebral endplate cancellous bone were packed into zero-profile interbody fusion cages. These cages were precisely implanted at the C3/4 and C4/5 levels, each secured with two self-locking screws. Final fluoroscopic verification confirmed optimal implant alignment. The surgical field was thoroughly irrigated, hemostasis was obtained, a closed suction drain was placed, and layered wound closure was performed.

### Postoperative evaluation

**Figure 2:**
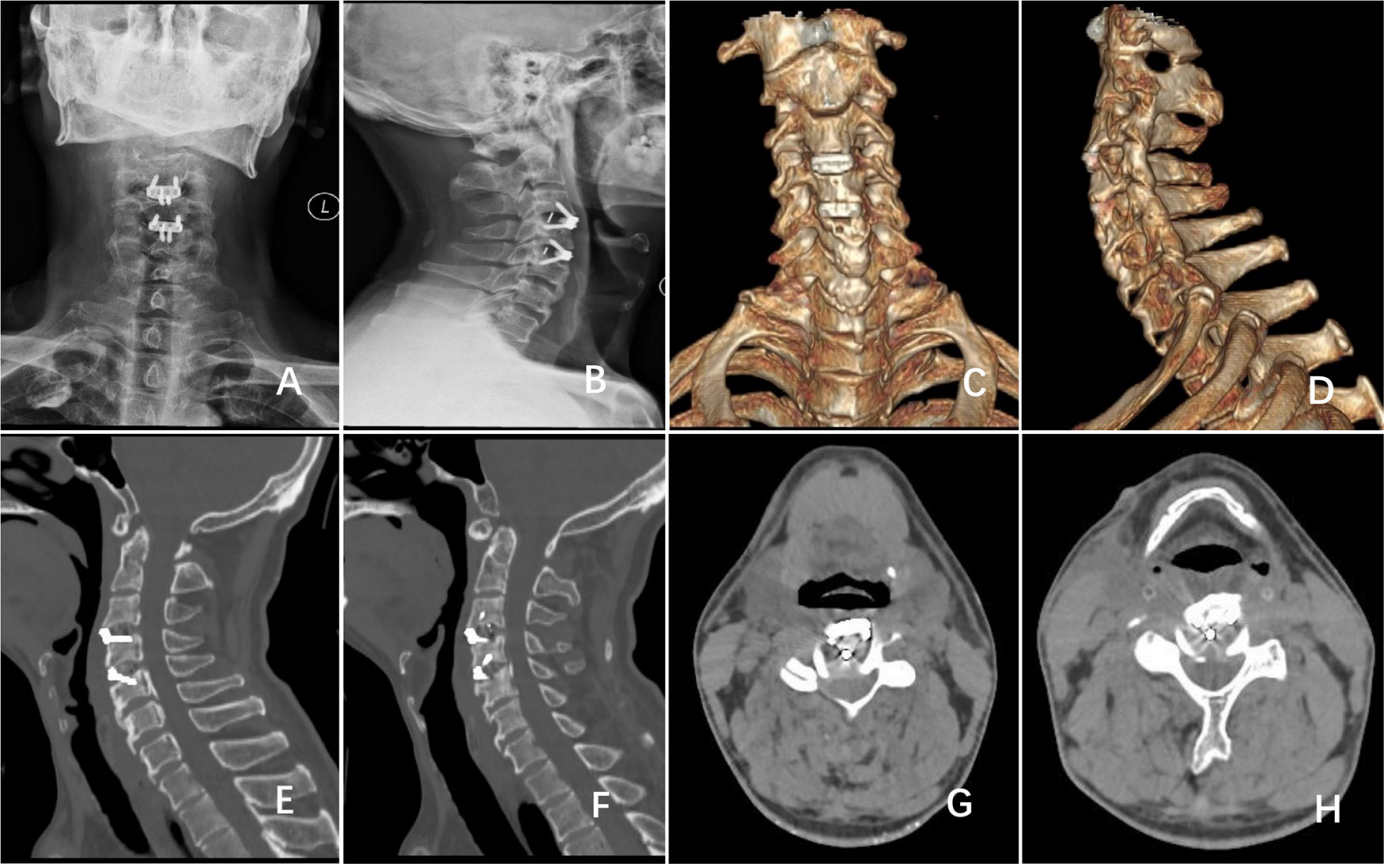
Postoperative follow-up **(A,B)** anteroposterior and lateral cervical x-rays and **(C,D)** cervical 3D-CT reconstruction demonstrated complete resection of anterior osteophytes at the C4 and C5 vertebral bodies, with zero-profile implants in place at the C3/4 and C4/5 intervertebral spaces. **(E–H)** Cervical CT revealed C3/4 and C4/5 disc herniation and partial resection of the posterior longitudinal ligament.

Postoperative evaluation revealed enhanced sensory perception, muscular strength, and motor coordination in all extremities. Functional outcomes improved to a JOA score of 15, NDI of 40% (moderate disability), and FOIS level 6. Postoperative imaging studies, including cervical radiographs (Figures 2A,B), CT scans (Figures 2E–H), and three-dimensional CT reconstructions (Figures 2C,D), confirmed complete anterior osteophyte resection, effective decompression of ossified posterior longitudinal ligament, adequate removal of herniated disc material, and biomechanically stable implant positioning. Although the patient declined postoperative cervical MRI for detailed spinal cord assessment, high-resolution thin-slice CT with multiplanar reconstruction demonstrated restored spinal canal anatomy and thecal sac contour, indirectly supporting successful neural decompression. Given CT's limited soft tissue resolution for evaluating spinal cord microstructural changes or vascular recovery, subsequent follow-up prioritized serial neurological evaluations using standardized scales to monitor functional progression.

### Two-month follow-up

**Figure 3:**
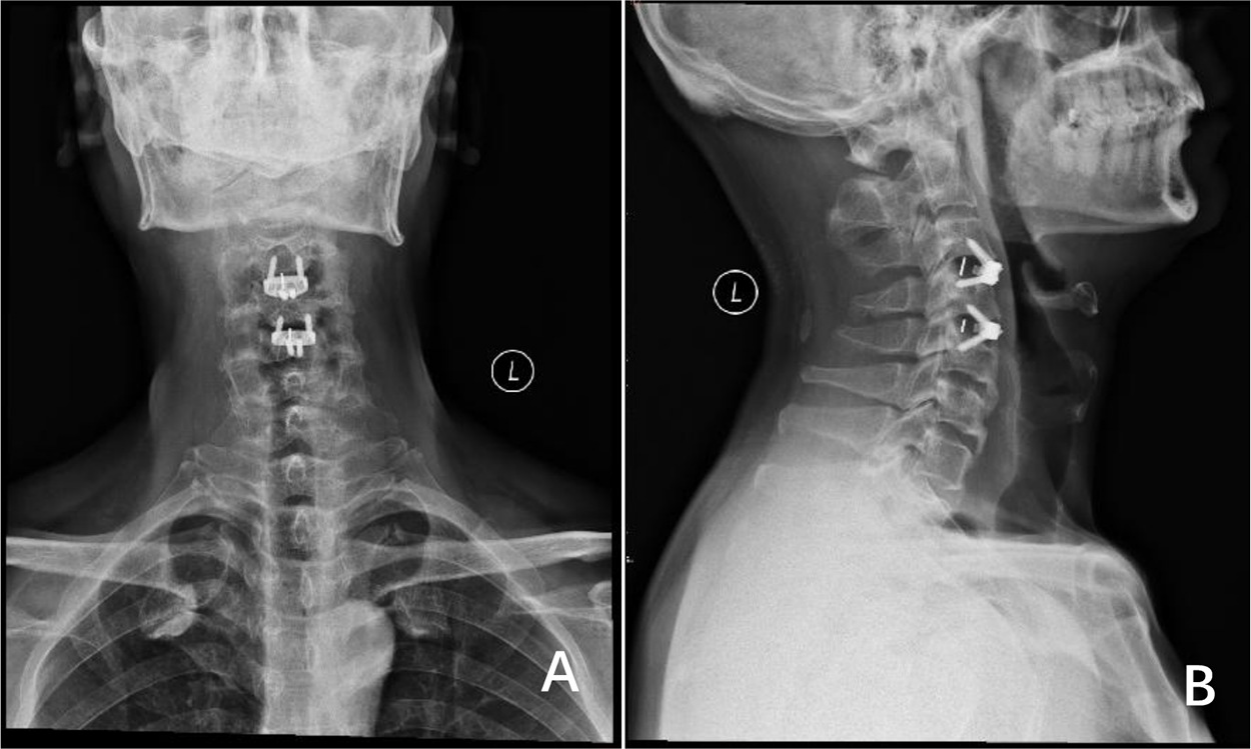
**(A,B)** anteroposterior and lateral x-ray views of the cervical spine demonstrate no recurrence of osteophytes at the C4 and C5 vertebral bodies, with no displacement of the zero-profile internal fixation devices.

At the two-month postoperative interval, cervical radiographs (Figures 3A,B) confirmed solid osseous fusion at C3/4 and C4/5 levels without osteophyte recurrence. Implant stability was evidenced by maintained physiological cervical curvature, preserved intervertebral height, and absence of radiolucent zones at screw-bone interfaces. The patient reported complete resolution of dysphagia with normal dietary intake, significant alleviation of bilateral lower extremity weakness, reduced “cotton-like” sensory symptoms, improved ambulatory capacity, and absence of pathological reflexes. Objective examination demonstrated full recovery of lower limb muscle strength (grade V). Functional metrics further improved to a JOA score of 17, NDI of 16% (mild disability), and FOIS level 7, consistent with favorable surgical outcomes.

## Discussion

Radiological evaluation demonstrated anterior osteophytes with bridging ossification at C4–6 vertebral levels, forming a localized osseous protrusion directly compressing the retropharyngeal space. These findings coexisted with OPLL spanning C3–7 and multilevel intervertebral disc herniation from C3/4 to C6/7, collectively constituting the characteristic degenerative “tetrad”: osteophytosis, ligamentous calcification, disc degeneration, and segmental instability. This pathological confluence generated dual compressive mechanisms: the prominent C4–5 anterior osteophyte chronically impinged on the posterior esophageal wall, where repetitive swallowing-induced friction risked mucosal inflammation or ulceration, while OPLL and herniated discs occupied the ventral spinal canal, exposing the cord to static compression (bony encroachment) and dynamic compression (ligamentous infolding during cervical motion). Additionally, ligamentum nuchae calcification suggested chronic biomechanical stress, emphasizing the necessity of restoring spinal stability during surgical intervention.

Diffuse idiopathic skeletal hyperostosis (DISH), a non-inflammatory disorder characterized by spinal ligament and entheseal calcification, is diagnosed radiographically using Resnick criteria requiring flowing anterolateral calcification across ≥4 contiguous vertebrae (4). Hallmark features include anterior longitudinal ligament ossification, predominantly in the thoracic spine (5). Imaging modalities such as radiography, CT, and MRI remain critical for assessing DISH severity (5). Differential diagnosis must exclude spinal osteoarthritis and ankylosing spondylitis, distinguished by absent sacroiliitis (vs. ankylosing spondylitis) (5) and preserved bone density (vs. metabolic bone diseases) (6). In this case, contiguous anterior ligament calcification from C4–7 (Figure 1H) confirmed DISH coexistence with disc herniation and OPLL.

Currently, there is a lack of clear epidemiological data on the coexistence of CSD and CSM. Existing research predominantly focuses on exploring the pathological mechanisms and optimizing treatment strategies for individual conditions. While studies on single pathologies still provide valuable insights for managing complex cases, there remains a significant research imbalance: CSD-related studies are relatively scarce compared to the well-established evidence-based system for CSM surgical treatment. This disparity highlights the need for cautious integration of surgical experiences from both conditions, combined with personalized assessments when formulating treatment plans for coexisting cases.

For severe CSD refractory to conservative treatment, anterior osteophyte resection combined with selective intervertebral fusion demonstrates significant efficacy. Surgical strategy selection depends on osteophyte distribution patterns and cervical stability assessments. In a case of non-contiguous osteophytes (C3/4 and C6/7 dual-level compression), osteophyte resection with two-level non-contiguous Zero-Profile fusion effectively relieved dysphagia while maintaining cervical stability through precise segmental fixation, with complete symptom resolution and no recurrence reported at 9-month follow-up (7). For a case of continuous multilevel lesions (C4–6 anterior giant osteophytes with disc herniation compressing the nerve root), three-level continuous ACDF achieved complete osteophyte removal and simultaneous disc pathology management, though postoperative outcome data require further validation (8). A clinical study of 14 patients (2009–2015) confirmed the long-term safety of this approach, showing significant swallowing improvement with no osteophyte recurrence or cervical instability during average 50-month follow-up. Notably, three cases required supplemental anterior plate fixation due to intraoperative segmental instability, emphasizing the importance of dynamic stability assessment (9). However, current evidence has limitations: small sample sizes in case reports, insufficient follow-up duration, and lack of evaluation for adjacent segment degeneration risk after multilevel fusion. Large-scale prospective studies are needed to clarify surgical indications and long-term outcomes across different techniques.

The surgical management of CSM necessitates a comprehensive assessment of compression topography, involved spinal segments, and biomechanical stability. Anterior surgical strategies, including ACDF and anterior cervical corpectomy and fusion (ACCF), serve distinct yet complementary roles. ACDF is preferentially employed for single- or multilevel disc herniation or focal osteophytic compression (10, 11), offering advantages in cervical alignment correction (12, 13) and lower complication rates (operative time, blood loss, and overall complication incidence compared to ACCF) (14, 15), though with potential accelerated adjacent segment degeneration (12, 13). In contrast, ACCF facilitates extensive decompression via vertebral body resection, demonstrating particular utility in multivertebral pathologies or severe structural compromise (16). While achieving more complete neural decompression (16), this technique carries elevated procedural complexity and complication risks (17). Consequently, ACDF remains the principal anterior approach (18), with ACCF reserved for cases requiring broad ventral decompression (19). Posterior approach selection is guided by stability requirements: laminoplasty preserves segmental mobility through expansive canal enlargement, optimally suited for multilevel OPLL with preserved spinal alignment (20–22), whereas laminectomy with instrumented fusion addresses kyphotic deformity or instability through posterior column stabilization (23–25). For CSM patients, through corresponding surgical treatment, mild-to-moderate cases (especially those with better preoperative neurological function) can achieve favorable neurological recovery (26), while severe cases, despite increased surgical risks, can still attain significant functional improvement (27).

The rationale for selecting a right anterior approach for osteophytectomy combined with two-level ACDF (C3/4, C4/5) includes the following considerations: (1) The C4–5 anterior osteophyte, exhibiting the largest bridging formation, constituted the primary pathological substrate for dysphagia, while concomitant C3/4 and C4/5 disc herniations with OPLL generated critical ventral spinal cord compression. (2) Conservative management of C5/6 and C6/7 disc herniations with mild cord compression aimed to preserve segmental mobility at these levels, potentially mitigating adjacent segment degeneration (ASD) risk through reduced iatrogenic alterations in load distribution and range of motion associated with additional fusion procedures (28–30). (3) The retained C6–7 anterior osteophytes and multilevel OPLL provided partial fusion-like stabilization, potentially slowing degenerative progression.

The two-level ACDF (C3/4, C4/5) with zero-profile interbody fusion devices—innovative implants integrating interbody fusion with low-profile fixation, obviating anterior plating—offered distinct advantages over traditional plate-screw systems. This design minimizes posterior esophageal irritation, significantly reducing postoperative dysphagia incidence (31), while demonstrating lower rates of adjacent level ossification development (ALOD), ASD, and hardware loosening (32). Biomechanically, these devices optimize cervical lordosis restoration and intervertebral height maintenance (33). Technical challenges arose during high cervical (C3/4) implantation due to mandibular interference limiting screw trajectory, suggesting future instrumentation modifications for enhanced anatomical adaptability. Preservation of C4/5–C5/6 osteophytes and OPLL was intentional, leveraging their stabilizing effects analogous to spontaneous fusion.

The rationale for foregoing laminoplasty despite extensive C3–7 OPLL included predominant ventral compression from disc pathology rather than continuous OPLL bridges, coupled with adequate spinal canal reserve space achieved through anterior decompression. Long-term surveillance remains crucial to validate anterior-only decompression efficacy in multilevel hybrid pathologies. Future advancements may integrate 3D-printed anatomical modeling for preoperative osteophyte resection simulation and cage positioning optimization, complemented by finite element analysis to predict ASD patterns and refine biomechanical surgical planning.

## Data Availability

The raw data supporting the conclusions of this article will be made available by the authors, without undue reservation.
